# Prevalence and Genetic Characterization of *Cryptosporidium* Species in Dairy Calves in Central Ethiopia

**DOI:** 10.1371/journal.pone.0154647

**Published:** 2016-05-02

**Authors:** Teklu Wegayehu, Robiul Karim, Manyazewal Anberber, Haileeyesus Adamu, Berhanu Erko, Longxian Zhang, Getachew Tilahun

**Affiliations:** 1 Aklilu Lemma Institute of Pathobiology, Addis Ababa University, Addis Ababa, Ethiopia; 2 Collage of Natural Sciences, Arba Minch University, Arba Minch, Ethiopia; 3 College of Veterinary Medicine, Addis Ababa University, Debre Zeit, Ethiopia; 4 Institute of Biotechnology, Addis Ababa University, Addis Ababa, Ethiopia; 5 Zhongshan School of Medicine, Sun Yat-sen University, Guangzhou, China; 6 College of Animal Sciences and Veterinary Medicine, Henan Agricultural University, Zhengzhou, Henan, China; Instituto de Higiene e Medicina Tropical, PORTUGAL

## Abstract

The burden of cryptosporidiosis due to *Cryptosporidium parvum* is well documented in HIV-positive patients in Ethiopia. However, the role of animals in zoonotic transmission of the disease is poorly understood. The aim of this study was to determine the prevalence and genotypes of *Cryptosporidium* species in dairy calves; to assess the role of cattle in zoonotic transmission in central Ethiopia. A total of 449 fecal samples were collected and screened using modified Ziehl-Neelson staining method and PCR targeting the small-subunit (SSU) rRNA gene. The prevalence of *Cryptosporidium* was 9.4% (42/449) and 15.8% (71/449) as detected by microscopy and nested PCR, respectively. The prevalence of infection varied significantly across the study areas with the higher prevalence being observed in Chancho 25.4% (30/118). Crossbred calves had significantly higher prevalence of *Cryptosporidium* than indigenous zebu. Genotyping results revealed the presence of *C*. *andersoni* (76.1%), *C*. *bovis* (19.7%) and *C*. *ryanae* (4.2%). The occurrence of these *Cryptosporidium* species appeared to be age-related. *C*. *andersoni* constituted 92.1% of the *Cryptosporidium* infection in calves older than 3 months. Sequence analysis also showed the existence of intra-species variation at SSU rRNA gene. Findings of the current study indicate that cattle may not be an important source of zoonotic cryptosporidiosis in central Ethiopia. Further molecular studies are needed to support this observation from other part of the country.

## Introduction

Cryptosporidiosis is predominantly a gastrointestinal disease of humans and other animals, caused by various species of protozoan parasites representing the genus *Cryptosporidium* [1]. Once thought to be a parasite of veterinary importance, *Cryptosporidium* has emerged as an important human pathogen, especially in pediatric, geriatric and immune-compromised patients where a fulminating infection may be life threatening [2]. Although human can get infection through several routes [3], the zoonotic transmission with respect to contact with cattle and other livestock has generated considerable interest in the epidemiology of the disease [4–6]. The vast majority of human cases are caused by *Cryptosporidium parvum* (*C*. *parvum*) and *C*. *hominis* [7,8].

Studies have indicated that cattle are commonly infected with four major *Cryptosporidium* species, namely: *C*. *parvum*, *C*. *bovis*, *C*. *andersoni* and *C*. *ryanae* [9–16]. The occurrence of the four species in cattle has shown to be age-related [9, 13, 17]. Thus, the zoonotic species (*C*. *parvum*) is mainly found in pre-weaned calves. *C*. *bovis* and *C*. *ryanae* usually infect weaned calves with *C*. *bovis* being more prevalent than *C*. *ryanae*. On the other hand, *C*. *andersoni* is commonly seen in yearlings and adult cattle. In addition, sporadic infection with *C*. *felis*, *C*. *hominis*, *C*. *suis*, *C*. *suis*-like genotype and *Cryptosporidium* pig genotype II have been reported in cattle [18–21].

The widespread use of molecular diagnostic tools has enabled us to understand the existence of genetic variation within *Cryptosporidium*. So far, more than 26 named *Cryptosporidium* species and over 70 genotypes have been described [3, 22]. Based on 60-kDa glycoprotein (gp60) gene data, at least 10 subtype families of *C*. *parvum* and numerous subtypes have been described from humans and other mammals [3, 23]. Of the 10 described *C*. *parvum* gp60 subtype families, IIa and IId have been reported from both human and non-human hosts and are found to be zoonotic [23].

The role of cattle in the zoonotic transmission of cryptosporidiosis is poorly understood in Ethiopia. This is due to the fact that studies on cryptosporidiosis in cattle were based on microscopy [24, 25] which does not enable accurate identification of the species encountered. Conversely, molecular studies conducted in humans identified *C*. *parvum* zoonotic subtype family IIa as the major cause of human cryptosporidiosis and suggest a zoonotic transmission of the disease in country [26, 27]. Therefore, the objective of the present study was to identify the species of *Cryptosporidium* present in dairy calves; and to assess their role in zoonotic cycle of the disease.

## Materials and Methods

### Study area

This cross-sectional study was conducted in Oromia Special Zone, central Ethiopia between January and June 2014. The Special Zone has an estimated 4,800 km^2^ which accounts for 1.5% of the total area of the Oromia Regional State. The zone borders with the capital, Addis Ababa, in all directions. Based on the available climatological data, the mean annual rainfall varies from 700 mm to 1400 mm in lowlands and highlands, respectively. The mean annual temperature of the Zone ranges between 20–25°C in the lowlands and 10–15°C in the highlands. The Special Zone has six districts and eight major towns of which Holetta, Sendafa and Chancho were selected for this study because of existence of large dairy farms and cattle populations.

### The study animals

The target population of this study was indigenous zebu and crossbred (Holstein Friesian X indigenous zebu) dairy calves. The indigenous zebu was widespread and owned by farmers in small number, on average 5 cattle per household. The crossbred, on the other hand, was mainly owned by private smallholder dairy farms and stratified into three categories based on the herd size as: small (<100), medium (100–200) and large (>200) (25). The weaning age of the indigenous zebu and crossbred was also different. The majority (85%) of the sampled calves were pre-weaned. A total of 449 dairy calves, age younger than five months, were included in this study.

### Specimen collection

Fresh fecal specimen was collected from each calf in sterilized stool container. The specimens were taken directly from the rectum of each calf or immediately after defecation using disposable gloves. Identification number, sex, age and breed of calves were recorded during sample collection. The specimens were transported to the Animal Health Parasitology Laboratory, Aklilu Lemma Institute of Pathobiology (ALIPB) at ambient temperature using cool box. A portion of the specimens was used for microscopy and the remaining was preserved with 2.5% potassium dichromate solution in 1:1 ratio and stored at 4°C prior to DNA extraction.

### Microscopy

Thin smears were prepared from sediments of formol-ether concentrated stool samples and stained with modified Ziehl-Neelson staining method [28]. Briefly, air-dried thin smears were fixed with absolute methanol for 5 minutes, air-dried and stained with carbol-fuchsin for 30 minutes. Smears were washed with tap water and decolorized with 1% acid-alcohol (1 ml HCl and 99 ml of 96% ethanol) for 2 minutes; washed with tap water and counterstained with 1% methylene blue for another 2 minutes, rinsed again in tap water and air-dried. The stained smears were examined by microscope using an oil immersion objective to screen oocysts of *Cryptosporidium*.

### DNA extraction

The preserved fecal specimens were washed with deionized water until the potassium dichromate was removed. Genomic DNA was extracted from each fecal sample using the E.Z.N.A.^®^ Stool DNA kit (Omega Biotek Inc., Norcross, USA). Briefly, about 50–100 mg of fecal specimen was added in a 2 ml centrifuge tube containing 200 mg of glass beads and placed on ice. Following, 300 μl buffer SP1 and proteinase K were added into the above mix, and incubated at 70°C for 10 minutes. Subsequently, all the procedures outline in product manual were performed according to the manufacturer’s protocol. Finally, DNA was eluted in 200 μl of elution buffer and the extract was stored at -20°C until PCR.

### Genotyping of *Cryptosporidium*

An approximately 830 bp fragment of the small-subunit (SSU) rRNA gene of *Cryptosporidium* was amplified by nested PCR as previously described [29]. The PCR was done in a 25 μl reaction volume containing 24 μl mixes and 1 μl DNA template. Amplification was performed using an Applied Biosystems Thermal Cycler version 2.09. The reaction mixture was initially incubated at 94°C for 5 minutes for initial denaturation and then a total of 35 reaction cycles were performed with denaturation at 94°C for 45 seconds, primer annealing at 55°C for 45 seconds and strand extension at 72°C for 1 minute. The final extension was done at 72°C for 10 minutes followed by cooling at 4°C.

The secondary PCR reactions were similar to the primary PCR with the exception that 2 μl of the primary PCR product was used as a template. In addition, the annealing temperature was raised from 55°C to 58°C for 45 seconds. Both positive and negative controls were included in each round of PCR to validate results. The amplified products were separated by electrophoresis on a 1% agarose gel and visualized under a trans-illuminator after staining with ethidium bromide. The PCR was conducted at the International Joint Research Laboratory for Zoonotic Diseases at Henan Agricultural University, China.

### DNA sequence analysis

All positive PCR products were purified using Montage PCR filters (Millipore, Bedford, MA) and sequenced using an ABI BigDye Terminator v. 3.1 cycle sequencing kit (Applied Biosystems, Foster City, CA) on an ABI 3100 automated sequencer (Applied Biosystems). Sequence accuracy was confirmed by sequencing both directions with primers used for the secondary PCRs. The raw nucleotide sequences and chromatograms of both forward and reverse directions were viewed using the EditSeq 5.0 and Chromas 2.4 programs, respectively. The nucleotide sequences were aligned and analyzed using ClustalX software. Consensus sequences were then compared to homologous sequences in GenBank using the Basic Local Alignment Search Tool (BLAST) (http://www.ncbi.nlm.nih.gov/blast/) to determine the species of *Cryptosporidium*. The nucleotide sequences generated in this study were deposited in the GenBank database under accession numbers: KT922228 to KT922235.

### Statistical analysis

Data were computerized using EpiData version 3.1 and transferred to STATA Software for analysis. Chi square test was used to verify possible association of *Cryptosporidium* infections with sex, age and breed of the calves. Values were considered to be statistically significant when the *p* value was < 0.05.

### Ethics Statement

Ethical clearance was obtained from the Institutional Review Board of ALIPB, Addis Ababa University and the National Health Research Ethics Review Committee. Support letters were obtained from Oromia Special Zone Animal Health Office and Agriculture Offices at community level. The objectives of the study were explained to owners of the calves before the collection of the specimens and permission was obtained.

## Results

From the total of 449 dairy calves, 153, 176 and 120 were sampled from Holetta, Sendafa and Chancho areas, respectively. Of these, 200 were male and 249 were female calves with male to female ratio of 1:1.2. The mean age of the calves were 2.5 months (ranged: 0.1 to 5 months). Furthermore, the calves were stratified in three age groups: < 1 month, 1–3 months and > 3 months making 113 (25.2%), 152 (33.9%) and 184 (41.0%) of the calves, respectively. The study calves were apparently healthy except few cases of watery diarrhea during sample collection.

### Prevalence of *Cryptosporidium*

Microscopic analysis using acid-fast staining showed that the prevalence of *Cryptosporidium* infection in calves was 9.4% (42/449). On the other hand, PCR analysis identified *Cryptosporidium* in 15.8% (71/449) (Table 1). All microscopy positive specimens were also found positive by PCR. The prevalence of *Cryptosporidium* detected by the two methods was significantly different (χ^2^ = 8.1532; *p* = 0.004).

**Table 1:** Prevalence of *Cryptosporidium* in dairy calves as detected by microscopy and PCR in central Ethiopia (January—June, 2014).

Study sites	No of samples examined	*Cryptosporidium*	
		Microscopy	Nested PCR
		No of samples positives (%)	No of samples positives (%)
Holetta	153	11 (7.2)	17 (11.1)
Sendafa	176	14 (7.9)	24 (13.6)
Chancho	120	17 (14.2)	30 (25.0)
**Total**	**449**	**42 (9.4)**	**71 (15.8)**

The PCR based prevalence of *Cryptosporidium* varied across the study areas with higher prevalence recorded in Chancho (25.0%) (Table 2). Similarly, as noted from Table 2, calves older than 3 months had a higher prevalence of *Cryptosporidium* (20.7%) than younger calves (*p* = 0.025). Likewise, the infection rate of *Cryptosporidium* in the crossbred (24.1%) was higher than that of the indigenous zebu (7.9%), and the difference was statistically significant (*p* = 0.00).

**Table 2:** PCR based prevalence of *Cryptosporidium* in dairy calves by study area, sex, age and breed groups in central Ethiopia (January—June, 2014).

Demographic characteristics	No of samples examined	*Cryptosporidium*		
		No of samples positives (%)	χ2	*p* value
**Study site**				
Holetta	153	17 (11.1)	10.77	0.005*
Sendafa	176	24 (13.6)		
Chancho	120	30 (25.0)		
**Sex**				
Male	200	29 (14.5)	0.467	0.494
Female	249	42 (16.9)		
**Age group**				
< 1 month	113	10 (8.8)	7.405	0.025*
1–3 months	152	23 (15.1)		
> 3 months	184	38 (20.7)		
**Breed group**				
Indigenous zebu	229	18 (7.9)	22.203	0.000*
Crossbred	220	53 (24.1)		

Key: *p* values compare the prevalence among study sites, sex, age and breed groups

Asterisks (*) Represent statistically significant difference

### Occurrence of *Cryptosporidium* species

The entire 71 samples which were positive by PCR amplification of SSU rRNA gene of *Cryptosporidium* were successfully sequenced. The genotyping results revealed the presence of three *Cryptosporidium* species, namely *C*. *andersoni*, *C*. *bovis* and *C*. *ryanae* (Table 3). *C*. *andersoni* was the most prevalent species being detected in 76.1% (54/71), followed by *C*. *bovis* 19.7% (14/71) and *C*. *ryanae* 4.2% (3/71). *C*. *parvum* was not detected in dairy calves in this study.

**Table 3:** Age-related distribution of *Cryptosporidium* species in calves in central Ethiopia (January—June, 2014).

Age group	No of samples positives	*Cryptosporidium* species occurrence		
		Number of species (%)		
		*C*. *andersoni*	*C*. *bovis*	*C*. *ryanae*
< 1 month	10	2(20.0)	7(70.0)	1(10.0)
1–3 months	23	17(74.0)	4(17.4)	2(8.7)
> 3 months	38	35(92.1)	3(7.9)	0(0.0)
**Total**	**71**	**54(76.1)**	**14(19.7)**	**3(4.2)**

There was age-related distribution of *Cryptosporidium* species among the calves (Table 3). *C*. *bovis* was the dominant species (70.0%) in calves younger than 1 month of age, whereas *C*. *andersoni* was the dominant species (92.1%) in calves older than 3 months. Although *C*. *ryanae* was occurred in calves younger than 3 months, the age-related distribution was not clear given that only three samples were positive.

### Genetic characterization

Sequence analysis showed intra-species variations at the SSU rRNA gene. Analysis of 54 *C*. *andersoni* isolates revealed the presence of three distinct variant copies of the gene, named as Type Ia, Type IIa, and Type IIIa for a convenient description (Table 4). Forty eight nucleotide sequences identified as Type Ia were identical to each other and had a 100% similarity with the reference sequence in the GenBank (accession number AB922119). Type IIa contained five isolates and showed 100% similarity to GenBank accession number KF826314. Type IIIa had 100% similar with the *C*. *andersoni* isolated from the dairy calves in Egypt (accession number AB513869). Both Type IIa and Type IIIa showed single nucleotide polymorphisms (SNPs) at position 431 and 57 respectively as compared to the reference AB922119.

**Table 4:** Intra-species variations at the SSU rRNA gene of *Cryptosporidium* species in central Ethiopia (January—June, 2014).

Type	GenBank accession no	No. of isolates	Nucleotide at position				
			32	57	69	407	431
***C*. *andersoni***							
Ref. seq.	AB922119		T	C	A	A	*
Type Ia	KT922228	48	*	*	*	*	*
Type IIa	KT922229	5	*	*	*	*	T
Type IIIa	KT922230	1	*	T	*	*	*
***C*. *bovis***							
Ref. seq.	JX515546		A	T	T	A	A
Type Ib	KT922231	9	*	*	*	*	*
Type IIb	KT922232	5	G	*	*	*	*
***C*. *ryanae***							
Ref. seq.	HQ179574		A	T	C	A	A
Type I	KT922234	1	*	*	*	*	*
Type II	KT922235	1	*	*	*	T	*
Type III	KT922233	1	*	*	T	*	*

Key: Asterisks (*) represent nucleotide identity to the reference sequences

Sequence analysis also revealed the presence of two distinct gene variant of *C*. *bovis*, named as Type Ib and Type IIb (Table 4). Of 14 nucleotide sequences identified as *C*. *bovis*, 9 were Type Ib and 100% identical to nucleotide sequences derived from dairy calves from China (GenBank Accession number JX515546). Five sequences assigned as Type IIb were 100% identical to isolates deposited in GenBank with accession number KF128742, but had one SNP as compared to the reference sequence.

Three of *C*. *ryanae* gene variants, Type I, Type II and Type III, were 100% similar to isolates of dairy calves with reference sequences deposited in the GenBank with accession numbers HQ179574, JX515550 and KP793011 respectively. Both Type II and Type III showed single SNP as compared to HQ179574 (Table 4).

## Discussion

The present study determines the prevalence and genotypes of *Cryptosporidium* species among dairy calves in three districts in Oromia Special Zone, central Ethiopia. Fecal specimens collected from calves were screened by microscopy using Ziehl-Neelson staining technique and nested PCR. Although microscopy is cheaper to perform and only method to indicates active infection, higher prevalence of *Cryptosporidium* was recorded by the PCR in this study. The superior sensitivity of PCR in detecting *Cryptosporidium* infection has been shown earlier clinical trial [30] and in patients from Northern India and South Africa [31, 32]. Thus, repeated microscopic examinations could reduce the discrepancy between PCR and microscopy in clinical setup.

The overall prevalence of bovine cryptosporidiosis in diary calves in this study was 15.8%. This finding was comparable with the 17.6% infection prevalence reported from selected dairy farms in central Ethiopia [25] and 16.0% reported from Kaduna State, Nigeria [33]. However, the prevalence was higher than the 7.8% reported from cattle of different age groups in Ethiopia by Wegayehu *et al*. [24] and 6.3% reported from traditionally reared calves younger than 3 months in Zambia by Geurden *et al*. [21]. On the other hand, the infection rate was lower than the 23.4% reported in 3–6 months age cattle in South Western Nigeria [34]. The disparities could be attributed to differences in management systems, age and breed of cattle, the sampling method and sample size, as well as diagnostic techniques employed in different study localities.

The prevalence of *Cryptosporidium* infection was significantly varied across the investigation areas. The highest prevalence of *Cryptosporidium* infection (25.0%) was recorded in Chancho. Although, many of the risk factors such as age, nursing conditions, husbandry and management system were roughly similar in the study areas, the hygienic conditions inside and around the farms might be responsible for the observed differences in the prevalence. Some of these factors may act individually or collectively to increase the risk factors associated with transmission and prevalence of *Cryptosporidium* infection between calves [35].

The prevalence of *Cryptosporidium* infection in crossbred was significantly higher than the indigenous zebu. This could be explained by the fact that management differences observed between the two breeds. One of the observed differences is that the crossbred was managed in farms in large herd size than the indigenous zebu. An association between large herd size and the risk of infection with *Cryptosporidium* has been reported in earlier studies [36, 37]. Large herds may have a heavier pathogen load because of increased density of animals that favors infection of great number of calves, which in turn, contaminate their surrounding environment.

The results of the current molecular study showed the presence of three major *Cryptosporidium* species in calves. The most prevalent was *C*. *andersoni* (76.1%). Our finding was in agreement with the previous report from central Ethiopia in dairy calves where Abebe *et al*. (25) presumed the species to be *C*. *andersoni*. The finding of the present study was also in agreement with the results from India [38, 39], the Czech Republic [40], Japan [41], Viet Nam [42] and Denmark [43]. *C*. *andersoni* was also reported as the second most prevalent species in pre-weaned calves in China (16). However, it was identified as less prevalent species in calves younger than 5 months in other studies [9, 33, 35, 44, 45]. The other two *Cryptosporidium* species identified from the calves were *C*. *bovis* and *C*. *ryanae* with *C*. *bovis* being more prevalent. The occurrence of these two species in dairy calves was well documented in previous studies [10–16, 45].

The zoonotic species, *C*. *parvum*, was not detected in calves irrespective of the age of calves in the present study. The absence of *C*. *parvum* has also been reported in early study conducted on agents associated with neonatal diarrhea in dairy farms in Ethiopia [46]. This was in agreement with the situation in Nigeria [33, 34], Zambia [21] and Japan [47]. Our findings suggest that cattle may not be important in the epidemiology of human cryptosporidiosis in Ethiopia. However, most of the earlier reports from different parts of the world [9, 11, 12, 15, 45] showed that *C*. *parvum* was the predominant species in pre-weaned calves which is in contrast to the present findings. The accurate reason for the absence of *C*. *parvum* remains unclear. Further molecular studies are needed to clarify the role of calves in epidemiology cryptosporidiosis in the country.

In this study, age-associated occurrence of *Cryptosporidium* species was seen in calves. Unlike the distribution in developed countries where *C*. *parvum* was mainly associated with calves younger than 2 months of age [9, 13], there was an early occurrence of *C*. *bovis* and *C*. *ryanae* in calves younger than 3 months. The finding was consistent with the previous report in native breeds of cattle in Kaduna State, Nigeria [33]. In addition, all five genetically characterized specimens from traditionally reared calves younger than three months in Zambia also belonged to *C*. *bovis* [21]. This findings might support the notion that in cattle reared in traditional husbandry systems in developing countries, *C*. *bovis* and *C*. *ryanae* appear early in calves in the absence of *C*. *parvum*.

*C*. *andersoni* is more frequently found in yearling and adult cattle [9, 11, 20]. However, the occurrence of *C*. *andersoni* in pre-weaned calves was also demonstrated [48]. In addition, an increase in occurrence of *C*. *andersoni* with increase in age of the animals was reported in previous studies in dairy and beef cattle [9, 13]. In our study, it was seen that there is a steadily increase in prevalence beginning from 20.0% in calves younger than 1 month, to 74.0% in calves age between 1–3 months, and thereafter 92.1% in calves older than 3 months. The highest prevalence of *C*. *andersoni*, though with a similar age related trend, was reported from the Czech Republic [48].

DNA sequence analysis also revealed intra-species variations within *C*. *andersoni*, *C*. *bovis* and *C*. *ryanae* at the partial SSU rRNA gene. Three distinct variant copies of the gene were obtained from 54 *C*. *andersoni* isolates with Type Ia being more prevalent. Likewise, one of the two distinct gene variant of *C*. *bovis* identified from 14 isolates, Type Ib was more prevalent. Similar intra-species variations were recently reported in *C*. *bovis* by Zhang *et al*. (16) in pre-weaned dairy calves in Northeastern China, Heilongjiang Province. Although three of the *C*. *ryanae* isolates showed 100% similarity to sequences deposited in the GenBank database, they had SNPs at positions 69 and 407. The findings might show genetic diversity within *Cryptosporidium* species in dairy calves in the study areas. To the best of our knowledge, this is the first molecular report of cryptosporidiosis in dairy calves from Ethiopia.

In conclusion, the results of the present study have provided useful information on the epidemiology of bovine cryptosporidiosis in central Ethiopia. Sequence analysis revealed the presence of *C*. *andersoni*, *C*. *bovis* and *C*. *ryanae* infection in varying magnitudes in the study area. The absence of the zoonotic species, *C*. *parvum*, in the calves suggests that cattle may not be important in the epidemiology of human cryptosporidiosis in the country. Nevertheless, this observation needs supports from additional studies from multiple regions, preferably involving larger sample size for a better understanding of the epidemiology of cryptosporidiosis in Ethiopia.
